# The Occurrence of Aflatoxins in Nuts and Dry Nuts Packed in Four Different Plastic Packaging from the Romanian Market

**DOI:** 10.3390/microorganisms9010061

**Published:** 2020-12-28

**Authors:** Adrian Maximilian Macri, Ioana Pop, Daniel Simeanu, Diana Toma, Ion Sandu, Liliana Lacramioara Pavel, Olimpia Smaranda Mintas

**Affiliations:** 1 Department of Animal Production and Food Safety, Faculty of Veterinary Medicine, University of Agricultural Sciences and Veterinary Medicine Cluj-Napoca, 3-5 Calea Manastur Street, 400372 Cluj-Napoca, Romania; adrian.macri@usamvcluj.ro; 2 Department of Land Measurements and Exact Sciences, Faculty of Horticulture, University of Agricultural Sciences and Veterinary Medicine Cluj-Napoca, 3-5 Calea Manastur Street, 400372 Cluj-Napoca, Romania; popioana@usamvcluj.ro; 3 Department of Fundamental Sciences in Animal Husbandry, Faculty of Animal Sciences, Ion Ionescu de la Brad University of Agricultural Sciences and Veterinary Medicine of Iasi, 3 Mihail Sadoveanu Alley, 700490 Iasi, Romania; 4 Department of Biochemistry and Biotechnology of Agri-Food Products, Faculty of Food Science and Technology, University of Agricultural Sciences and Veterinary Medicine Cluj-Napoca, 64 Calea Floresti Street, 400509 Cluj-Napoca, Romania; diana.toma@usamvcluj.ro; 5 Academy of Romanian Scientists (AOSR), 54 Splaiul Independentei St., Sect. 5, 050094 Bucharest, Romania; 6 Department of Conservation of Cultural Heritage, Institute of Interdisciplinary Research, Alexandru Ioan Cuza University of Iasi, ARHEOINVEST Centrum, 11 Carol I, Bld., 700506 Iasi, Romania; 7 Romanian Inventors Forum, Str. Sf. P. Movila 3, 700089 Iasi, Romania; 8 Department of Forensic Medicine, Faculty of Medicine and Pharmacy, Dunarea de Jos University of Galati, Str. Domneasca, nr. 47, 800008 Galati, Romania; doctorpavel2012@yahoo.ro; 9 Department of Animal Science and Agritourism, Faculty of Environmental Protection, University of Oradea, 1 University Street, 410087 Oradea, Romania; buzasiu@yahoo.com

**Keywords:** mycotoxins, aflatoxin B_1_, nuts, plastic packaging, ELISA, immunoaffinity

## Abstract

Mycotoxins are secondary metabolites produced by various fungi. A very important category of mycotoxins are aflatoxins, considered to be the most dangerous in humans. Aflatoxin B_1_, well known as a favorable factor in the occurrence of hepatocellular carcinoma in humans, is the most controversial of all mycotoxins. Aflatoxins, found in naturally contaminated food, are resistant to degradation by heat. Current food processing practices and conventional storage conditions do not completely eliminate aflatoxin contamination from the food supply chain. Long storage food products—such as peanuts, pistachio, nuts in general, and dried fruits—are susceptible to aflatoxins contamination. The type of plastic material can influence the concentration of aflatoxins during storage due to the permeability to gas and moisture exchange with the external milieu. Nuts in general and dried fruits are consumed in large quantities worldwide. Therefore, herein we investigated the effect of plastic material on the total aflatoxins and aflatoxin B_1_ content in 64 samples of nuts and dried fruits packed and stored in low-density polyethylene (LDPE), polypropylene (PP), polyethylene (PE), and polyethylene terephthalate (PET). The method consisted in a cleanup procedure using immunoaffinity columns coupled with RIDASCREEN FAST immunoenzymatic competitive assays based on the ELISA technique. Collected data were subjected to statistical analysis and multiple comparisons tests were applied. From the total analyzed samples, 14.06% exceeded the maximum admitted European levels for total aflatoxins. The highest concentrations of total aflatoxins were obtained from samples packed in LDPE, followed by PP, PE, and PET. Aflatoxin B_1_ was detected in all samples packed in LDPE, PP, and PE. Most of the samples packed in PET had concentrations <1 µg/kg. These results indicate that nuts in general packed and stored in LDPE are more prone to contamination with aflatoxins, while PET is more suitable for maintaining the quality and safety of these products.

## 1. Introduction

Mycotoxins are secondary metabolites with low molecular weight, produced by various fungi that are able to grow on different agricultural commodities. Consumption of contaminated food with mycotoxins leads to adverse effects on human health, such as carcinogenic, estrogenic, neurotoxic, hepatotoxic, teratogenic, and even immunosuppressive effects, further causing acute or chronic diseases [1,2,3,4,5]. Great attention has been paid to the aflatoxin-producing fungi belonging to the *Aspergillus flavus* and *Aspergillus parasiticus* species due to their ability to grow on a variety of food matrices [6] and to synthesize aflatoxins, which are some of the most dangerous mycotoxins [7]. Aflatoxin B_1_ (AfB_1_) is considered by the International Agency for Research on Cancer (IARC) to be the most carcinogenic compound produced by fungi [8]. In humans, a large body of evidence shows that hepatocellular carcinoma can be induced by aflatoxins. Behind the carcinogenicity of aflatoxins stands a complex genotoxic mechanism of action that involves metabolite activation to a genotoxic epoxide metabolite, together with DNA adducts formation and the modification of the *TP53* gene. Up to 50% of the patients diagnosed with hepatocellular carcinoma (reported from regions where population exposure to aflatoxins is high) display a specific point mutation in the *TP53* tumor-suppressor gene [9]. Considering the negative impact of aflatoxins on human health, worldwide organizations have set maximum admitted levels for different food types [10,11,12].

Fungal production of mycotoxins can occur at any stage of the food production process, both pre- and postharvesting, and can expose consumers to the risk of contamination directly through food consumption [13]. Generally speaking, mold growth and mycotoxin production depend on several factors, among which the most important are substrate moisture content, relative air humidity, and ambient temperature [14]. In the case of cold storage warehouses, temperatures should be maintained within the range of 1–5 °C. As regards the relative air humidity, this should be between 55% and 70%. When oleaginous products are removed from cold storage, this should be done tempered, in order to allow the temperature to reach 18 °C. This prevents the formation of condensate moisture; therefore, it reduces the risk of mold growth. Most preventive practices for aflatoxin formation rely mainly on the avoidance of postharvest contamination based on fast drying techniques and good storage practices [15].

In particular, long storage food products—such as dried fruits, peanuts, and other tree nuts—are susceptible to aflatoxins contamination [16,17,18,19,20,21]. However, likewise is the case of ogi and fufu, two dietary staples obtained through a homemade maize fermentation process and widely consumed by the African population [22,23]. Recently, a complete screening of samples of pearl millet intended for human consumption prospected from different Tunisian areas was performed, and the results revealed a higher prevalence of aflatoxin B_1_ [24]. According to another study completed on commonly used spices in Iranian cuisine, spices may also contain serious amounts of aflatoxins. Additionally, the concentrations recorded were in the order of red chili > garam masala > black pepper > turmeric [25]. Mycotoxin contamination was also observed in Mediterranean dried figs, fruits susceptible to unavoidable contaminants such as *Aspergillus*, considered dominant fungi [26]. Aflatoxin contamination occurs even in the components of complementary foods for infants and young children in Nigeria—in the form of infant formulas, milk, nut- and cereal-based foods. As such, young children at the weaning age may be exposed through consumption of both household-formulated and industrially processed contaminated complementary foods [27,28]. Human exposure to aflatoxins from foods naturally contaminated was assessed by a biomonitoring point of view through biomarkers present in urine and breast milk samples [29,30]. Although low in concentration, aflatoxin traces were discovered even in blood samples of women and children. This demonstrates once more that the safety of consumers is at high risk [31].

Lately, a major postharvest challenge concerning the shelf life and quality of food products, including oleaginous products, is represented by the use of proper packaging materials. Although the most important role of food packaging is to maintain quality and food safety, these criteria are difficult to meet, especially for plastic packaging [32,33]. The positive side of plastic packaging is that, due to its transparency, it allows ultraviolet (UV) light passage, followed by a small reduction of aflatoxins contamination [34]. On the other hand, visible light (VIS) could promote the growth of *A. flavus* and can trigger higher aflatoxin production [35]. Since fungal attack usually begins in the outer parts of the seed, mycotoxin contamination tends to be greater here than on the inside part. However, both scenarios regarding light radiation are possible, together with storage conditions, crop chemical composition, and the chemical properties of the mycotoxin [36]. Since plastic is not an inert material (like glass), in addition to the undesirable migration of toxic chemicals from plastic into food [2,37], the type of plastic material can promote indirectly the toxicity of the aflatoxins during storage if it is permeable to gas and moisture coming from the environment [38]. When nitrogen is used as a filling gas during food packaging, this has a positive influence on the quality and storage life of pearl millet-based fried snacks [39]. Peanut kernels artificially inoculated with aflatoxigenic *A. flavus* and *A. parasiticus*, packed and stored for one month in different types of plastic packaging, have shown differences in AfB_1_ content. The highest concentration of AfB_1_ was detected in low-density polyethylene (LDPE) for both *A. flavus* (46.41 ppb) and *A. parasiticus* (414.42 ppb), followed by polypropylene (PP) (*A. flavus* 24.29 ppb; *A. parasiticus* 386.73 ppb) [40].

The reduction or inactivation of aflatoxin content in nuts in general is very difficult or even impossible due to their resistance, even at the very high temperatures applied during food processing, such as roasting [41]. Although some physical, chemical, and biological control measures are available for controlling aflatoxin contamination, some of these are not suitable for food application and can make the product unwholesome for human consumption. Most of the chemical and physical approaches were proved to be efficient in the case of mycotoxins decontamination, but the majority of these methods are suitable more for feedstuffs. Such examples are sorbent additives, which act as binding agents to prevent AfB_1_ absorption from the intestinal tract after ingestion, or irradiation treatment on groundnuts [42].

Regarding the analysis of food samples containing aflatoxins, a cleanup step of the sample is an important process to eliminate those substances that may interfere with the subsequent detection of mycotoxins. By cleaning up the extract, the specificity and sensitivity are enhanced, resulting in improved accuracy and precision. Analysis of aflatoxins in different food and feed samples is nowadays performed by various methods, including thin-layer chromatography (TLC), high-performance liquid chromatography (HPLC), enzyme-linked immune sorbent assay (ELISA) [43] and the LC–MS/MS method using a dilute-and-shoot approach [44].

Globally, the production of tree nuts in general and dried fruits reached immense quantities in 2018. For example, 490,028 metric tons of hazelnuts and 135,400 metric tons for dried figs were produced, and production continues to expand annually [45]. Given that nuts in general and dried fruits are consumed in large quantities by the worldwide population, herein we studied the incidence of mycotoxins in these types of products. We also investigated the effect of plastic packaging materials on the level of total aflatoxins and aflatoxin B_1_, well known as favorable factors in the occurrence of hepatocellular carcinoma in humans.

## 2. Materials and Methods

### 2.1. Materials

Food matrices were represented by 64 samples of a number of different types of nuts and mixes packed in PET (polyethylene terephthalate), PP (polypropylene), LDPE (low-density polyethylene), and PE (polyethylene) bags from the Romanian market as follows: 4 samples of roasted pistachio, 4 samples of raw hazelnuts, 1 sample of Brazil nuts, 6 samples of walnut, 1 of apricot kernels, 5 of roasted almonds, 1 of Macadamia nuts, 5 of fruits and nuts mix, 2 of raw peanuts, 5 of roasted corn, 1 of popcorn, 4 of dehydrated fruits, 3 of roasted peanuts in the shell, 7 of roasted peanuts, 5 of raw cashews, 3 of roasted hazelnuts, 3 of roasted American peanuts, 2 of roasted cashews, and 2 of raw almonds. The weight of each sample purchased from the market varied between 100–250 g.

The laboratory equipment used for this study was: laboratory balance (Kern & SOHN GmbH, Balingen, Germany), laboratory mill (RETSCH GmbH, Haan, Germany), microplates shaker (Antisel, Romania), magnetic stirrer, fluted paper filter, one-way plastic syringes, graduated pipettes, micropipettes, and glassware.

For the quantitative determinations of total aflatoxins and AfB_1_, we utilized the laboratory kits RIDASCREEN FAST Aflatoxin and RIDASCREEN FAST Aflatoxin B_1_ (R-Biopharm AG, Darmstadt, Germany), respectively. The primary scope of the Aflatoxin kits was to determine the mycotoxin concentration in feedstuffs, but the method of combining the aflatoxin kits and an immunoaffinity column was validated and described elsewhere [46]. The total aflatoxin determination kit contained: 1× microplate (6 strips of 8 wells each, removable), 48 wells lined with antibodies, and 5× aflatoxin standard solutions, 1.0 mL each, with concentrations of 0 µg/kg (zero standard), 1.7 µg/kg, 5 µg/kg, 15 µg/kg, and 45 µg/kg aflatoxin in water/methanol; 1× anti-aflatoxin antibody (3 mL), ready to use; 1× conjugate (3 mL), ready to use; 1× substrate/chromogen (6 mL); 1× stop reagent (6 mL) and 1 M sulfuric acid; and 1× washing solution. The AfB_1_ determination kit contained: 1× microplate ELISA with 96 removable wells; 6× aflatoxin standards (1.3 mL each) of 0 µg/kg, 1 µg/kg, 5 µg/kg, 10 µg/kg, 20 µg/kg, and 50 µg/kg; 1× conjugate (6 mL); 1× anti- aflatoxin antibody (6 mL); 1× substrate/chromogen (10 mL); 1× stop solution (14 mL); 1× washing solution (salt). Prior to analysis of total aflatoxins and aflatoxin B_1_, a single-use immunoaffinity RIDA Aflatoxin column (R-Biopharm AG, Darmstadt, Germany) was used for sample cleanup. In addition to the kits and immunoaffinity columns, methanol (Merck, ≥99.99%), Tween 20 (Merck, ultrapure), and distilled water were used.

### 2.2. Methods

Aflatoxins are light-sensitive; therefore, the exposure of the samples, the sample extracts, and the kits to direct light was avoided before the preparation steps. First of all, samples were ground as finely as possible to produce a homogeneous particle size by means of a laboratory mill.

All 64 samples were subjected to determinations for total aflatoxins and AfB_1_. Samples were divided into 4 experimental groups based on their type of packaging as follows: LDPE group with 15 samples, PE group with 15 samples, PP group with 15 samples, and PET group with 19 samples.

Of each finely ground sample, 5 g were weighed and placed in a flask together with 25 mL of 70% methanol. The extraction was facilitated by mixing with a magnetic stirrer over a 10 min period. The obtained extract was filtered through a fluted paper filter. Furthermore, 5 mL from the filtered extract was combined with 15 mL distilled water and the obtained mixture (20 mL) was passed entirely over an immunoaffinity column.

Before use, each immunoaffinity column was placed at room temperature. The columns were plugged with caps on the top and tip, which were removed prior to use. Initially, the column was rinsed with 2 mL of distilled water to remove the storage buffer above the gel. The column was filled with 1 mL of sample solution that was prepared beforehand. A suitable adapter was attached on top of the column and a syringe was used as a sample reservoir. The sample was passed slowly and continuously through the column, respecting a flow rate of approximatively 1 drop/sec, in order to prevent compression of the gel and, consequently, the possible loss of aflatoxins. After the passed solution was discarded, the column was rinsed with 10 mL of distilled water, and again the passed solution was discarded. Some air was introduced through the column to make sure that all the residual fluids were removed from the column. Subsequently, the syringe was removed. A clean and closable vial was placed directly below the column. A volume of 0.5 mL of pure methanol was passed slowly through the column to ensure the complete elution of the aflatoxins. When the eluent passed too fast, this step was repeated. All eluent residues were collected by pressing air thoroughly through the column.

Sample preparation with the immunoaffinity columns simplified and enhanced the sample cleanup procedure. By cleaning up the extract, the specificity and sensitivity were enhanced, resulting in improved accuracy and precision. Pure extracts were obtained based on the antigen–antibody reaction. The column contained a gel suspension to which monoclonal antibodies were attached covalently. The antibodies were specific for the aflatoxins B_1_, B_2_, G_1_, and G_2_. After the sample was applied and was passed through the column, the aflatoxins present in the sample were bound by the monoclonal antibodies. All other substances were not retained on the column. Using methanol as eluent, the aflatoxins were released from the antigen–antibody complex due to denaturation of the antibodies caused by the methanol. Therefore, the antigen (aflatoxin) was set free and was further eluted.

The cleanup procedure was followed by the determination of total aflatoxin and AfB_1_. For this purpose, 50 μL of toxin containing eluent (sample resulted after the cleanup process) was diluted with 450 μL of distilled water.

Of each solution, 50 µL was used for testing total aflatoxins, and another 50 µL was used for AfB_1_ determination. After the preparation of the samples, a sufficient number of well strips were inserted in support. The position of the standards and samples was recorded, after which 50 µL standard solutions was added to the samples in separate wells; pipette tips were changed after each sample. After adding standard solutions, 50 μL of the enzymatic conjugate was added to each well, followed by 50 μL of aflatoxin antibody in each well. Wells were stirred and incubated for 10 min at room temperature (in the dark). After incubation, the liquid from the wells was thrown into the sink with an energetic movement and the plate was tapped face down on the filter paper to remove the liquid from the wells. To each well was added 250 µL of distilled water, and the liquid was discarded again. This step was repeated twice. To each well was added 100 μL of substrate/chromogen. It was stirred well and incubated for 5 min at room temperature (in the dark), after which 100 μL of stop reagent was added to each well. Both determination of the total aflatoxin and AfB_1_ followed the same procedural steps, except that 15 min were waited for AfB_1_ with the substrate/chromogen. The microplates were stirred well, and the absorbance was read at 450 nm with the aid of microplate reader ChroMate-4300 (Awareness Technology Inc, Palm City, FL, USA). The reading was performed within 10 min of the addition of the stop reagent using RIDA Soft software. The absorbance was inversely proportional to the amount of aflatoxin.

For quantification, the average absorbance value obtained for standards and samples was divided by the absorbance value of the first standard and multiplied by 100. The zero standard is equal to 100% and the absorbance values are in percent.

### 2.3. Statistical Analysis

Regarding the occurrence of total aflatoxins and AfB_1_ in samples packed in plastic materials, statistical tests were applied to differentiate the levels of these mycotoxins.

Collected data were subjected to statistical analysis using IBM SPSS Statistics 20 software. The normality of the groups was verified using the Shapiro–Wilk test. For the descriptive statistics, the mean values and the standard deviations for the normally distributed data, as well as medians and percentiles (25–75) for data that were not normally distributed, were determined.

One-way ANOVA (analysis of variance) with post hoc Tukey HSD (honestly significant difference) tests were used to make multiple comparisons. The Scheffé, Bonferroni, and Holm tests were also used to strengthen the results obtained with post hoc Turkey HSD tests.

## 3. Results and Discussions

From the total of 64 samples analyzed for total aflatoxin content, 60 (93.75%) were positive and 4 (6.25%) were below the detection limit (<1 µg/kg) for aflatoxins. Out of the positive samples, 9 (14.06%) exceeded the maximum level admitted by regulation CE Reg. No. 1881/2006 and Commission Regulation (EC) No 165/2010 [47]. The samples that exceeded the tolerated values of total aflatoxins were: 3 samples of fruits and nuts mix with the highest value of 12.46 µg/kg (maximum admitted value 10 µg/kg) from the LDPE group, 1 sample of roasted corn with 7.81 µg/kg (maximum admitted value 4 µg/kg) from the LDPE group, 1 sample of raw hazelnuts with 12.15 µg/kg (maximum admitted value 10 µg/kg) from the LDPE group, 1 sample of apricot kernels with 10.67 µg/kg (maximum admitted value of 10 µg/kg) from the LDPE group, 1 sample of raw peanuts with 8.22 µg/kg (maximum admitted value of 4 µg/kg) from the LDPE group, 1 sample of raw peanuts with 4.4 µg/kg from the PE group, and 1 sample of roasted corn with 5.04 µg/kg from the PP group. Concentrations found in samples from the PP and PE groups were different. For instance, the PP group had a maximum value of 7.36 µg/kg, whereas the maximum value from the PE group was 4.4 µg/kg.

**Figure 1:**
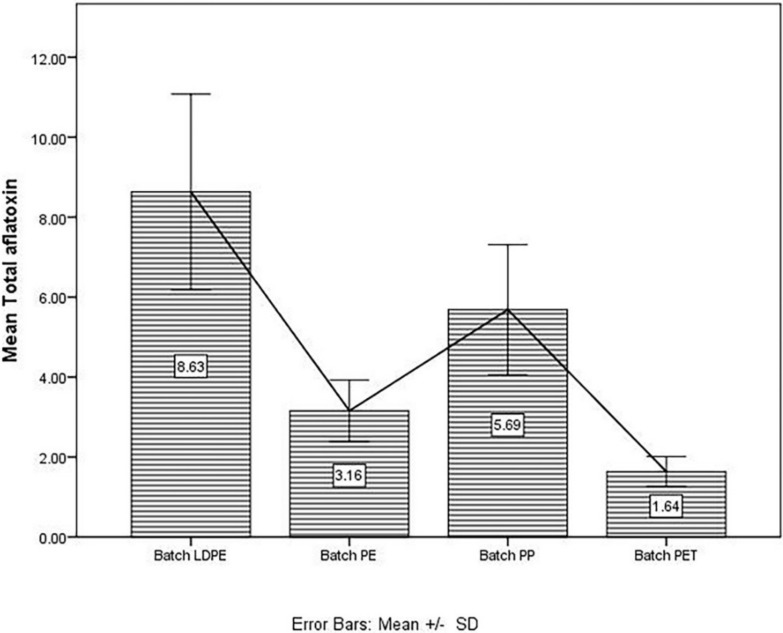
Mean concentration of total aflatoxins (µg/kg) and calculated standard deviation corresponding to each batch of screened samples.

The highest value from the PET group for total aflatoxins was obtained from a sample of raw almonds, i.e., 2.13 µg/kg, and was much below the maximum admitted level of 10 µg/kg. Among the tree nuts, pistachio seems to be more prone to aflatoxins contamination, and peanuts are likewise. The pistachio sample packed in an LDPE bag turned out to have a total aflatoxins content of 8.25 µg/kg, followed by 6.16 µg/kg for pistachio stored in a PP bag and by 1.79 µg/kg for pistachio stored in PET material. The mean concentrations of total aflatoxins from the screened samples ranged from 1.64 µg/kg to 8.64 µg/kg and can be observed in Figure 1. The highest mean value for total aflatoxins corresponds to the LDPE group; the lowest mean concentration of total aflatoxins corresponds to the PET group.

The concentrations of AfB_1_ from all screened samples had lower values than the maximum limits imposed by legislation, except for one sample of raw hazelnut from the LDPE group, which had a value of 5.78 µg/kg. Nuts packed in LDPE had the highest detected mean levels of aflatoxins. The concentrations of AfB_1_ detected in samples from the PET group were mostly below the detection limit of <1 µg/kg; only 3 had values above the detection limit: roasted peanuts with paprika 1.1 µg/kg, fruits mix 1.09 µg/kg, and roasted corn 1.13 µg/kg. The maximum concentration for AfB_1_ found in the PP group was 3.64 µg/kg in a raw cashew sample, while the highest value from the PE group was registered in a sample of roasted peanuts, 1.73 µg/kg. Concentrations of 4.31 and 3.37 µg/kg of AfB_1_ were detected for dried nuts and fruits from the LDPE group, respectively. The AfB_1_ found in roasted peanut samples ranged from 1.02 µg/kg (PE group) to 1.77 µg/kg (PP group).

**Figure 2:**
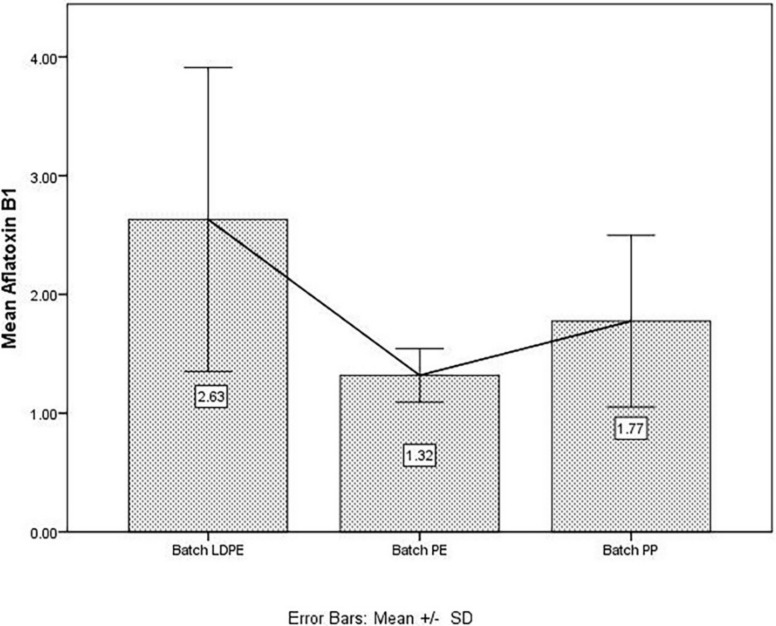
Mean concentration of Aflatoxin B_1_ (AfB1) (µg/kg) in low-density polyethylene (LDPE), polyethylene (PE), and polypropylene (PP) batches. SD: standard deviation calculated for each batch of screened sample.

With regard to the mean concentrations of AfB_1_ from the analyzed samples, the PE group presented the lowest mean values (Figure 2). As concerns the PET group, the data from this group were excluded from statistical analysis due to the fact that most of the concentrations were below the detection limit of 1 µg/kg.

Hepsag et al. detected total levels of aflatoxins in pistachio samples, ranging from 0.26 µg/kg to 385 µg/kg and from 0.16 to 60.9 µg/kg in groundnuts [48]. Fu et al., 2018 found in their study that PET is more suitable to store peanuts, pistachios, and other oleaginous products due to its good properties for maintaining the safety of the products [49,50].

If an infestation in nuts in general has naturally occurred before harvesting and if the *Aspergillus* molds were not eliminated during processing, these are able to further contaminate the food. Since these types of products are kept at room temperature, which sometimes/most of the time exceeds 20 °C, toxin production becomes possible because of the enforced temperatures ranging from 20 °C to 30 °C [51]. It was demonstrated that when stored in LDPE bags at room temperature for 10 months, pistachio suffered changes in moisture content, which led to a serious increase of total aflatoxins level of 1019.03 µg/kg, compared to an increase of up to 97.6 µg/kg in pistachio stored in PET for the same duration [52].

For instance, when inoculated with *Aspergillus flavus* and *A. parasiticus* and stored in LDPE bags, peanuts pose the highest concentration of AfB_1_ compared with PP [41].

The incidence of AfB_1_ in nuts samples was detected in a concentration that ranged between 0.48–36.81 µg/kg in pistachio, 0.22–33.4 µg/kg in peanuts, and 1.17–1.80 µg/kg in hazelnuts. These results were obtained during a study performed on various nuts commercialized in Turkey [53]. Several authors have also found that pistachio is prone to contamination with AfB_1_, with mean values of 8.9 µg/kg, followed by peanuts and corn [54], while some authors confirmed a concentration range between 2.66 and 8.85 µg/kg [5]. In a study performed in New Zealand, the mean value of AfB_1_ in roasted peanuts was 38 µg/kg [55,56].

All data from each group were included in statistical analysis, except 4 samples from PET group, which were below the detection limit (<1 µg/kg) for total aflatoxins. Following the descriptive statistics, the highest mean value for total aflatoxins came from the LDPE group samples (Table 1).

Applying the Shapiro–Wilk test for total aflatoxins, it was observed that the values of the LDPE group, PE group, PP group and PET group were normally distributed. The data, being parametric, were expressed as mean ± standard deviation (std dev).

For the total aflatoxins, the value *p* = 0.000, corresponding to an F = 60.0858 statistic for the one-way ANOVA, was less than 0.05, which suggests that there were significant differences between one or more groups.

Subsequently, analysis with multiple tests confirmed the differences between groups that resulted from the post hoc Tukey HSD (honestly significant difference) tests. The tests showed which of the pairs of groups were significantly different from each other. It was noted that the same findings were obtained after the application of each post hoc type of test (Table 2).

As concerns AfB_1_, the data from the PET group were not taken into consideration for any of the statistics due to values <1 µg/kg. As shown in Table 3, the highest mean value was registered in the LDPE group, as was the case for total aflatoxins. The minimum values of AfB_1_ were similar in all three experimental groups.

For the parametric data, means ± standard deviations (std devs) are included in the table, and for the nonparametric data, the median percentiles (25–75) are used. The value *p* = 0.000, corresponding to an F = 9.0304 statistic for the one-way ANOVA, was less than 0.05, which suggests that there were significant differences between one or more groups.

According to the Tukey HSD test, the value of *p* = 0.000 correlated with the F = 9.0304 statistic of the unidirectional ANOVA and was less than 0.01, which strongly suggests that one or more pairs of treatments were significantly different. The post hoc multiple tests identified the differences between the groups and are presented in Table 4.

When applied to aflatoxin B_1_ data, the Shapiro–Wilk test showed that the LDPE group and PE group were less normally distributed than the PP group.

## 4. Conclusions

To sum up, contamination with total aflatoxins was observed in 93.75% of the analyzed samples. Out of these, 14.06% exceeded the maximum permitted levels and could be declared nonconforming. Aflatoxin B_1_ was observed in 75% of the total screened samples, but only one sample of raw hazelnuts exceeded the maximum admitted levels. Both non-heat-treated (raw) and processed samples (roasted, seasoned) were contaminated with aflatoxins, suggesting that the thermal factor was not decisively influencing the decrease in aflatoxin content in the analyzed samples. Corn, pistachio, peanuts, and dried fruits were more prone to contamination.

Based on the obtained results we conclude that samples packed in PET significantly yielded the lowest AfB_1_ concentrations, followed by PE, PP, and LDPE. Likewise, was the case for total aflatoxins content in all analyzed samples. The detailed effects of the type of plastic packaging on the concentrations of detected aflatoxins still needs to be resolved.

## Figures and Tables

**Table 1:** Descriptive statistics for total aflatoxins (µg/kg).

Treatment	LDPE Group	PE Group	PP Group	PET Group
Observations (*n*)	15	15	15	15
Mean ± std dev.	8.63 ± 2.44	3.16 ± 0.77	5.69 ± 1.62	1.64 ± 0.37
Min-Max	3.45–12.46	2.15–4.40	2.79–7.63	1.14–2.13

(*n*)-number of samples from each group; std dev-standard deviation; Min-minimum; Max-maximum; LDPE-low-density polyethylene; PE-polyethylene; PP-polypropylene; PET-polyethylene terephthalate.

**Table 2:** Multiple comparisons based on advanced statistics post hoc analysis.

	Tukey HSD *p*-Value	Tukey HSD Inference	Scheffé *p*-Value	Scheffé Inference	Bonferroni *p*-Value	Bonferroni Inference	Holm *p*-Value	Holm Inference
LDPE vs. PE	0.001	** *p* < 0.01	0.000	** *p* < 0.01	0.000	** *p* < 0.01	0.000	** *p* < 0.01
LDPE vs. PP	0.001	** *p* < 0.01	0.000	** *p* < 0.01	0.000	** *p* < 0.01	0.000	** *p* < 0.01
LDPE vs. PET	0.001	** *p* < 0.01	0.000	** *p* < 0.01	0.000	** *p* < 0.01	0.000	** *p* < 0.01
PE vs. PP	0.001	** *p* < 0.01	0.000	** *p* < 0.01	0.000	** *p* < 0.01	0.000	** *p* < 0.01
PE vs. PET	0.041	* *p* < 0.05	0.071	*p* > 0.05	0.052	*p* > 0.05	0.000	** *p* < 0.01
PP vs. PET	0.001	** *p* < 0.01	0.000	** *p* < 0.01	0.000	** *p* < 0.01	0.000	** *p* < 0.01

** significant, *p* < 0.01; * significant, *p* < 0.05; HSD-honestly significant difference; LDPE-low-density polyethylene; PE-polyethylene; PP-polypropylene; PET-polyethylene terephthalate.

**Table 3:** Descriptive statistics for aflatoxin B1 (µg/kg).

Treatment	LDPE Group	PE Group	PP Group
Observations (*n*)	15	15	15
Mean ± std devMedian Percentile (25–75)	2.63 ± 1.27	1.32 ± 0.22	1.77 (1.18–2.11)
Min-Max	1.09–5.78	1.04–1.73	1.07–3.64

(*n*)-number of samples from each group; std dev-standard deviation; Min-minimum; Max-maximum; LDPE-low-density polyethylene; PE-polyethylene; PP-polypropylene; PET-polyethylene terephthalate.

**Table 4:** Advanced statistics post hoc analysis.

	Tukey HSD *p*-Value	Tukey HSD Inference	Scheffé *p*-Value	Scheffé Inference	Bonferroni *p*-Value	Bonferroni Inference	Holm *p*-Value	Holm Inference
LDPE vs. PE	0.001	** *p* < 0.01	0.000	** *p* < 0.01	0.000	** *p* < 0.01	0.000	** *p* < 0.01
LDPE vs. PP	0.024	* *p* < 0.05	0.032	* *p* < 0.05	0.027	* *p* < 0.05	0.018	* *p* < 0.05
PE vs. PP	0.323	*p* > 0.05	0.356	*p* > 0.05	0.459	*p* > 0.05	0.153	*p* > 0.05

* significant, *p* < 0.05; ** significant, *p* < 0.01; HSD-honestly significant difference; LDPE-low-density polyethylene; PE-polyethylene; PP-polypropylene; PET-polyethylene terephthalate.

## Data Availability

The data presented in this study are available on request from the corresponding authors.
